# Synaptic Integration in CA1 Pyramidal Neurons Is Intact despite Deficits in GABAergic Transmission in the *Scn1a* Haploinsufficiency Mouse Model of Dravet Syndrome

**DOI:** 10.1523/ENEURO.0080-22.2022

**Published:** 2022-05-16

**Authors:** Jessica Hotard Chancey, MacKenzie Allen Howard

**Affiliations:** 1 Department of Neurology, Dell Medical School, Austin 78712, TX; 2 Department of Neuroscience and Center for Learning and Memory, University of Texas at Austin, Austin 78712, TX

**Keywords:** Dravet syndrome, epilepsy, mouse models, *Scn1a*, synaptic integration, synaptic transmission

## Abstract

Mutations of *SCN1A*, which encodes the voltage-gated sodium channel Na_v_1.1, can cause epilepsy disorders such as Dravet syndrome (DS) that are comorbid with wide-ranging neurologic dysfunction. Many studies suggest that Na_v_1.1 haploinsufficiency causes forebrain GABAergic interneuron hypoexcitability, while pyramidal neuron physiology is mostly unaltered, and that this serves as a primary cell physiology phenotype linking mutation to disease. We hypothesized that deficits in inhibition would alter synaptic integration during activation of the hippocampal microcircuit, thus disrupting cellular information processing and leading to seizures and cognitive deficits. We tested this hypothesis using *ex vivo* whole-cell recordings from CA1 pyramidal neurons in a heterozygous *Scn1a* knock-out mouse model and wild-type (WT) littermates, measuring responses to single and patterned synaptic stimulation and spontaneous synaptic activity. Overall, our experiments reveal a surprising normalcy of excitatory and inhibitory synaptic temporal integration in the hippocampus of *Scn1a* haploinsufficient mice. While miniature IPSCs and feedforward inhibition and were decreased, we did not identify a pattern or frequency of input that caused a failure of synaptic inhibition. We further show that reduced GABA release probability and subsequent reduced short-term depression may act to overcome deficits in inhibition normalizing input/output functions in the *Scn1a* haploinsufficient hippocampus. These experiments show that CA1 pyramidal neuron synaptic processing is surprisingly robust, even during decreased interneuron function, and more complex circuit activity is likely required to reveal altered function in the hippocampal microcircuit.

## Significance Statement

Mouse models of genetic epilepsy disorders are useful tools for better understanding the neurophysiology underlying the seizures and cognitive comorbidities of disease. Here, we use a *Scn1a* haploinsufficieny model of Dravet syndrome (DS) to investigate synaptic integration and input/output functions, which are fundamental forms of neuronal information processing. We found that while DS model hippocampal pyramidal neurons had modest deficits in inhibition, synaptic integration and input/output functions were surprisingly normal. We also found that changes in GABA release probability may be a compensatory mechanism by which the hippocampal circuit overcomes deficits in inhibition to normalize input/output functions.

## Introduction

Disruption of the most vital building blocks of neural signaling lead to the most severe neurologic outcomes. The *SCN1A* gene encodes one such foundational component of neuronal excitability, Na_v_1.1, a voltage-gated sodium channel α subunit that supports action potential generation in many neurons (Catterall et al., 2010). Mutations to *SCN1A* lead to epilepsy disorders ranging from simple febrile seizures to the spectrum of generalized epilepsy with febrile seizures plus (GEFS+) disorders (Catterall et al., 2010; Escayg and Goldin, 2010). Patients with a mutation that renders one allele of *SCN1A* nonfunctional often present with the most severe form of GEFS+: Dravet syndrome [DS; also known as severe myoclonic epilepsy in infancy (SMEI); Claes et al., 2001; Marini et al., 2011]. With limited treatment options, DS is a profoundly devastating disease characterized by frequent, severe seizures, developmental delays, a range of comorbid neurologic and cardiac deficits, and high mortality rates (Dravet, 2011).

The epilepsy, comorbidities, and high mortality exhibited by DS patients are recapitulated in genetically engineered animal models of *SCN1A* disruption, including rat (Mashimo et al., 2010), mouse (Yu et al., 2006; Mistry et al., 2014), zebrafish (Baraban et al., 2013), and *Drosophila* (L. Sun et al., 2012). Initial characterization of heterozygous *Scn1a* mice revealed decreased sodium currents and action potential generation in GABAergic interneurons in cortex and hippocampus, resulting in decreased synaptic inhibition (Yu et al., 2006). In contrast, sodium currents in pyramidal neurons were unaffected. Work in animal models across phyla, in heterozygous knock-out and human disease mutation knock-in models, and interneuron specific Na_v_1.1 knock-outs offer support to the hypothesis that decreased inhibitory neuron function is an electrophysiological signature of, and major mechanism underlying, neural dysfunction in *SCN1A*-linked epilepsy disorders (Catterall, 2018).

Further studies have added depth and complexity to the interneuron-dysfunction hypothesis of DS. *In vivo* spike rates of interneurons have been recorded to be normal or even elevated during ongoing activity in heterozygous mice (De Stasi et al., 2016; Tran et al., 2020). Further, the decreased intrinsic excitability of parvalbumin-expressing and somatostatin-expressing interneurons is transient, normalizing to wild-type (WT) levels after a developmental delay (Favero et al., 2018; Almog et al., 2021). This suggests that other developmental abnormalities or compensatory changes in the neural circuitry persist beyond the recovery of function of these inhibitory neurons. A recent study found that while parvalbumin interneurons are hypoexcitable in the dentate gyrus of young adult *Scn1a*^+/−^ mice, the magnitude of evoked inhibition was modestly higher, not lower, in dentate granule cells in response to perforant path stimulation, and that excitatory/inhibitory ratio and granule cell recruitment did increase, but because of increases in excitatory input rather than deficits in inhibition (Mattis et al., 2022). Finally, vasoactive intestinal peptide (VIP)-expressing neurons, which largely target other GABAergic interneurons, and therefore disinhibit neural circuitry, also exhibit excitability deficits in heterozygous mice (Goff and Goldberg, 2019). Thus, the mechanism underlying DS is more complicated than simple circuit disinhibition because of GABAergic neuron hypoexcitability.

Neural circuit activity and information processing depend on the complex integration of temporally patterned excitatory and inhibitory synaptic inputs (Stuart and Spruston, 2015). We hypothesized that deficits in interneuron function would alter synaptic integration and synaptic input/action potential output functions in forebrain pyramidal neurons, serving as a circuit mechanism by which interneuron hypofunction causes cognitive processing deficits of DS. We tested this hypothesis by examining temporal synaptic integration and detailing excitatory and inhibitory synaptic physiology in CA1 hippocampal pyramidal neurons in *ex vivo* slices from *Scn1a* heterozygous and WT littermate mice. Despite clear evidence of severe epilepsy, broad neurologic dysfunction, and early death in the *Scn1a* deficient mouse model used here (Miller et al., 2014; Mistry et al., 2014; Tran et al., 2020), we found only subtle changes to synaptic inhibition, and overall, synaptic integration and input/output functions were relatively unchanged in the hippocampus of this DS model.

## Materials and Methods

### Animals

**Figure 1.**
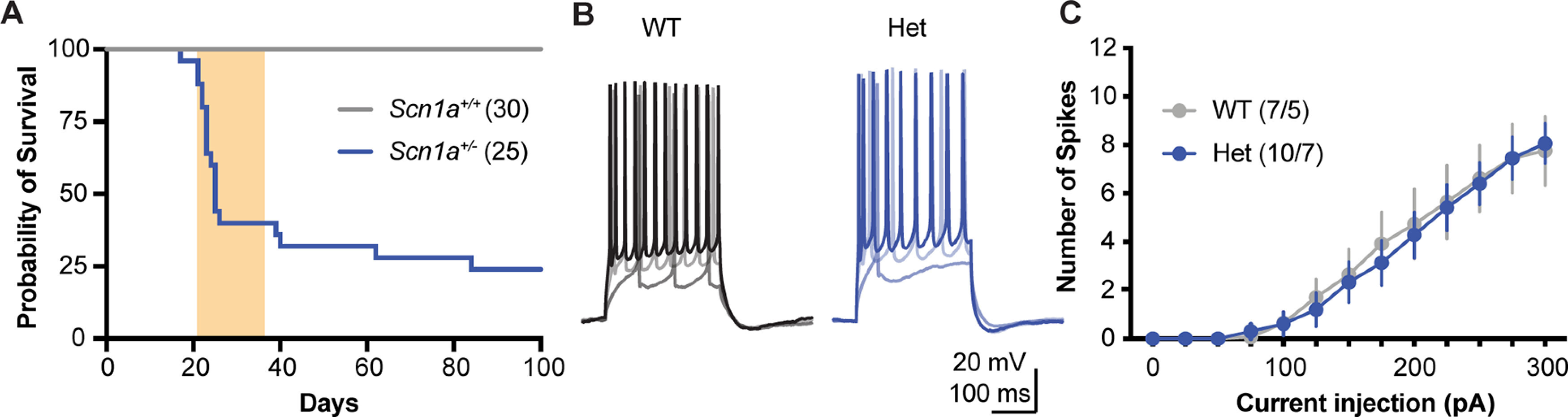
Verification of reported *Scn1a*^+/−^ phenotypes. ***A***, Survival plot of *Scn1a*^+/−^ (blue; *n* = 25) and *Scn1a^+/+^* WT litter mates (WT; gray; *n* = 30) from seven litters, plotted as probability of survival per day. ***p < 0.001, χ^2^ test. All electrophysiology experiments were done during the period shaded in orange (P21–P38). ***B***, Example whole-cell recordings of neuron membrane potential to depolarizing current steps (100-, 200-, and 300-pA steps). ***C***, Spike number plotted as a function of current step size (main effect of current step: *F*_(12,180)_ = 62.73; *p* < 0.0001; genotype: *F*_(1,15)_ = 0.03; *p* = 0.87; interaction: *F*_(12,180)_ = 0.16; *p* = 0.99). Error bars for this and all subsequent figures indicate standard error of the mean, n = (cells/mice).

All procedures were approved by the Institutional Animal Care and Use Committee at our institution. A breeding colony of *Scn1a*^+/−^ mice (a generous gift from Jennifer Kearney) was maintained on the 129S6/SvEvTac background strain (Taconic). Experimental animals were produced by crossing these mice with WT C57Bl/6J mice (The Jackson Laboratory, strain #000664). All experiments were performed on the 129.*Scn1a*^+/−^ x C56/B6J F1 generation as in Mistry et al. (2014). Experimental animals were male and female 129/C57 *Scn1a*^+/−^ (Het), while male and female WT 129/C57 *Scn1a^+/+^* littermates served as controls. Pups were toe-clipped at approximately postnatal day (P)7 for identification and tail tissue samples were taken for genotyping by PCR (Miller et al., 2014). All experiments were performed in mice age P21–P38 (Fig. 1*A*, shaded area). A subset of mice was maintained in their homecages and checked on daily for a Kaplan–Meier analysis of survival (Fig. 1*A*). Of the Het mice monitored (*n* = 25), the first incidence of mortality was recorded on P17, 50% died by P25, while 24% survived at least 100 d. No WT littermate deaths were recorded (*n* = 30).

### Acute slice preparation

Mice were injected intraperitoneally with a mix of ketamine (90 mg/kg) and xylazine (10 mg/kg). Once deep anesthesia was confirmed by lack of response to toe pinch, mice were transcardially perfused with ice-cold, oxygenated cutting solution, containing (in mm): 205 sucrose, 25 sodium bicarbonate, 2.5 KCl, 1.25 sodium phosphate, 7 MgCl_2_, 7 D-glucose, 3 sodium pyruvate, 1.3 ascorbic acid, and 0.5 CaCl_2_. Perfused brains were removed and hemisected. The midline was placed down and the dorsal portion of the brain was removed using a scalpel blade, angled at ∼30° (Poolos and Jones, 2004). The cut side was then mounted in the slicing chamber of a Leica VT1200 vibratome; 300-μm horizontal hippocampal slices were made from brains submerged in ice-cold, oxygenated cutting solution. Sections were then incubated for 30 min at 37°C, thereafter at room temperature, in holding solution containing (in mM): 125 NaCl, 25 sodium bicarbonate, 2.5 KCl, 1.25 sodium phosphate, 12.5 D-glucose, 2 MgCl_2_, 2 CaCl_2_, 1.3 ascorbic acid, and 3 sodium pyruvate.

### Whole-cell electrophysiology

Slices strictly from the intermediate region of the dorsal-ventral axis of the hippocampus were used, as pyramidal neuron intrinsic excitability, morphology, and synaptic physiology and plasticity all change along this axis (Dougherty et al., 2012; Malik et al., 2016; Milior et al., 2016). Slices were placed in a recording chamber and continuously bathed in warm (32°C), oxygenated artificial CSF (ACSF), containing (in mm): 125 NaCl, 25 NaHCO_3_, 2.5 KCl, 1.25 NaH_2_PO_4_, 1 MgCl_2_, and 2 CaCl_2_. For some experiments, synaptic receptors were pharmacologically blocked: GABA_A_ receptors with gabazine (gbz, 10 μm), AMPA receptors with NBQX (10 μm), NMDA receptors with APV (50 μm) in the ACSF. Patch pipettes were pulled to resistances of 3–6 MΩ. Pipettes were filled with either potassium gluconate-based or cesium-based solutions. Potassium gluconate internal contained (in mm): 120 K-gluconate, 20 KCl, 10 HEPES, 4 NaCl, 1 EGTA, 4 Mg-ATP, 0.3 Na-GTP, and 7 phosphocreatine disodium salt hydrate. Cesium methanesulfonate internal contained (in mm): 120 CsMeS, 4 NaCl, 6 CsCl, 10 HEPES, 1 EGTA, 4 Mg-ATP, 0.3 Na-GTP, 7 phosphocreatine disodium salt hydrate, and 5 QX-314. Cesium chloride based internal contained (in mm): 80 CsCl, 45 CsMeS, 4 NaCl, 6 CsCl, 10 HEPES, 1 EGTA, 4 Mg-ATP, 0.3 Na-GTP, 7 phosphocreatine disodium salt hydrate, and 5 QX-314. Slices were visualized using a Zeiss Axio Examiner, an infrared digital camera (Zeiss Axiocam 503) and Dodt contrast optics. Neurons with depolarized membrane potential (>−50 mV) or high series resistance (>25 MΩ) were excluded from the study. Synaptic stimulation via activation of the Schaffer collateral (SC) axons was performed using a bipolar stimulating electrode (Microprobes) placed in the stratum radiatum near the CA2/CA1 border and a constant current stimulus isolator (World Precision Instruments).

Data were acquired using a MultiClamp 700B amplifier (Molecular Devices) and digitized using an InstruTECH LIH8 + 8 (HEKA) at a sampling rate of 10 kHz, then filtered at 4 kHz. Data were acquired using Axograph acquisition software. After measuring resting membrane potential in current clamp experiments, a holding current was applied to give all neurons a baseline membrane potential of ∼−70 mV. Action potential input/output curves and voltage sag were measured using 250-ms-long depolarizing and hyperpolarizing current steps. Voltage sag was calculated as the peak negative voltage minus the steady state negative voltage just before the end of the hyperpolarizing step when cell was hyperpolarized to −90 mV. For synaptic stimulation experiments, threshold stimulus intensity was measured as the lowest stimulus power that consistently produced a measurable response.

### Data analysis and statistics

All data are presented as mean ± SEM. Data were analyzed using either Student’s *t* tests, Mann–Whitney tests, Kolmogorov–Smirnov (K-S) tests, or repeated measures (RM) ANOVA followed by *post hoc* Sidak’s multiple comparisons tests, as appropriate using GraphPad Prism 7.

## Results

### Firing properties of CA1 pyramidal neurons are normal in *Scn1a* Het mice

Previous studies have reported that pyramidal neurons in *Scn1a* haploinsufficiency mouse models have relatively normal intrinsic physiological properties (Hedrich et al., 2014; Rubinstein et al., 2015; Almog et al., 2021), while others have found increased intrinsic excitability (Mistry et al., 2014). Because multiple investigators have independently engineered *Scn1a* transgenic mice, our initial experiments served to establish measures of baseline excitability in CA1 hippocampal pyramidal neurons in this particular line in our hands. Whole-cell current clamp recordings showed that many standard electrophysiological measures were statistically indistinguishable in Het neurons compared with control, including resting membrane potential, input resistance, membrane time constant, capacitance, and voltage sag (Table 1).

**Table 1:** Intrinsic electrophysiological properties of CA1 pyramidal neurons

	*WT* (*n* = 7 cells from 5 mice)	*Het* (*n* = 10 cells from 7 mice)	*p* value (unpaired *t* test)
Resting membrane potential (mV)	−60.59 ± 1.83	−58.40 ± 1.61	0.385
Input resistance (MΩ)	136.60 ± 16.90	118.00 ± 10.34	0.335
Membrane time constant (ms)	17.19 ± 2.12	12.64 ± 6.06	0.138
Capacitance (pF)	132.00 ± 13.6	112.10 ± 18.00	0.428
Voltage sag (mV)	5.81 ± 0.52	6.72 ± 0.44	0.190
Rheobase (pA)	153.60 ± 24.7	140.00 ± 13.50	0.611
Voltage threshold (mV)	−39.22 ± 1.70	−38.25 ± 1.05	0.617
1st spike rise time (μs)	269.40 ± 21.21	255.50 ± 22.68	0.674
1st spike half width (ms)	1.49 ± 0.12	1.36 ± 0.09	0.375

Suprathreshold depolarizing current steps were used to evoke and quantify action potential parameters. Like previous reports in other *Scn1a* knock-out mouse lines (Hedrich et al., 2014; Dyment et al., 2020; Almog et al., 2021), action potentials were largely unchanged in Het neurons compared with littermate controls. This can be observed in quantification of number of spikes evoked as a function of current injection (WT *n* = 7; Het *n* = 10; two-way RM ANOVA: main effect of current *p* < 0.0001; main effect of genotype *p* = 0.87; interaction of current and genotype *p* = 0.99; Fig. 1*B*). Measures of rheobase, voltage threshold, first spike rise time and half width were also similar across genotypes (Table 1).

### Functional integration of excitation and inhibition are unchanged despite decrements in inhibition in Scn1a haploinsufficiency

Our central hypothesis was that decrements in GABAergic interneuron excitability caused by *Scn1a* haploinsufficiency would cause changes in the output of pyramidal neurons in response to activation of the circuit. We tested this hypothesis with *ex vivo* recordings from CA1 pyramidal neurons in current clamp, using a K-gluconate-based internal solution and stimulating the SC pathway.

**Figure 2.**
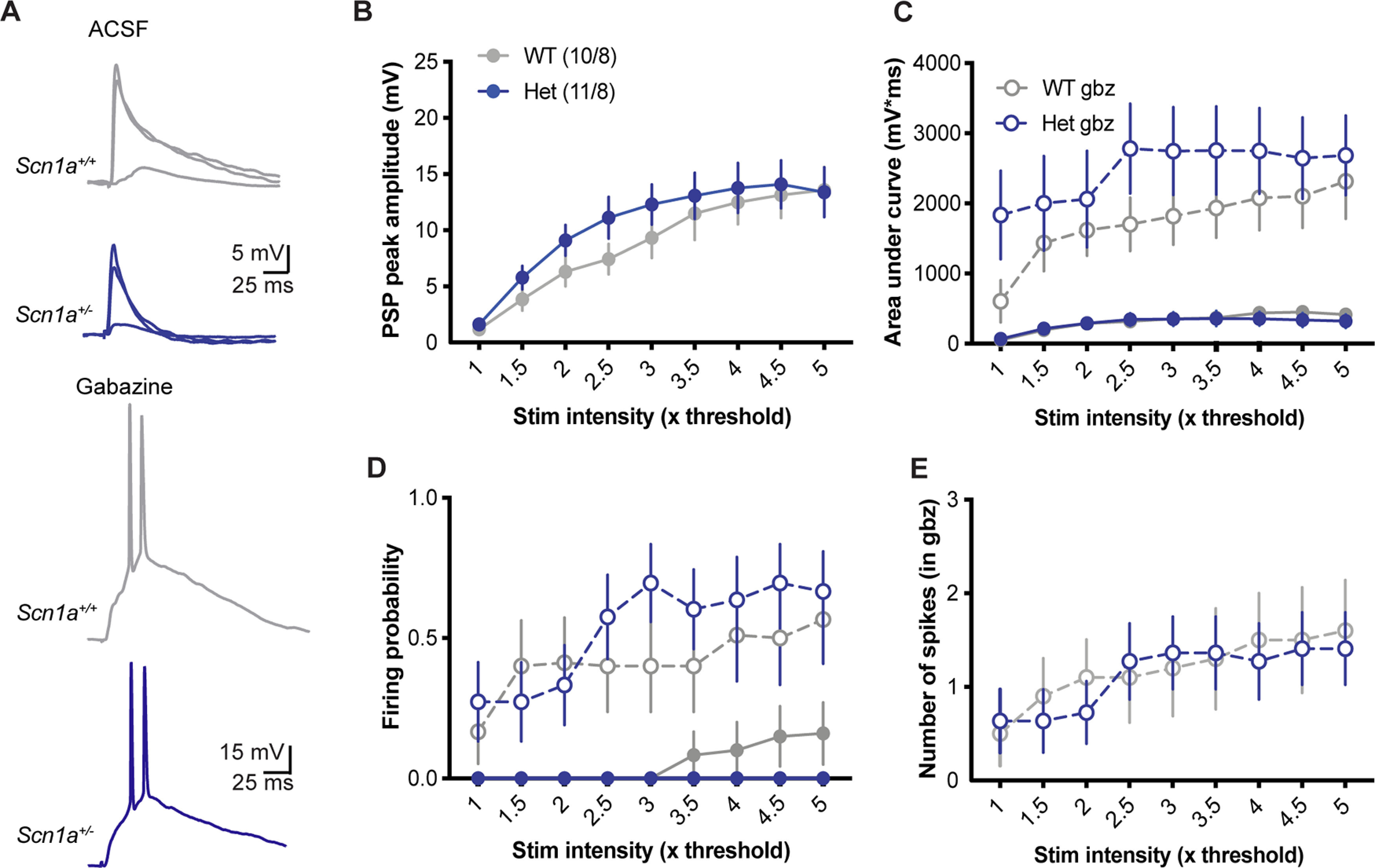
Similar PSPs in CA1 pyramidal neurons from WT and *Scn1a*^+/−^ mice. ***A***, Example PSPs recorded in CA1 pyramidal cells from *Scn1a^+/+^* (WT; gray) and *Scn1a*^+/−^ (Het; blue) mice in response to single stimulations of SC axons at 1×, 3×, and 5× the minimal stim intensity in ACSF (top) and 3× the minimal stim in 10 μm gabazine (gbz; bottom). ***B***, The peak amplitude across a range of stim intensities is similar between genotypes [main effect of genotype: *F*_(1,19)_ = 0.62; *p* = 0.44, main effect of stim intensity: *F*_(1.57,29.83)_ = 51.18; *p* < 0.0001, interaction: *F*_(8,152)_ = 1.11; *p* = 0.36; two-way RM ANOVA; *n* = (cells/mice)]. ***C***, Firing probability across stim intensities is similar between genotypes (main effect of genotype: *F*_(1,38)_ = 0.09; *p* = 0.77, main effect of stim intensity: *F*_(2.68,101.7)_ = 7.09; *p* < 0.001; three-way RM ANOVA) and is similarly increased by gbz application in both genotypes (main effect of drug: *F*_(1,38)_ = 21.98; *p* < 0.0001, genotype × drug: *F*_(1,38)_ = 0.76; *p* = 0.39). ***D***, Area under the curve, with spikes truncated, across stim intensities is similar between genotypes (main effect of genotype: *F*_(1,38)_ = 0.96; *p* = 0.33, main effect of stim intensity: *F*_(2.68,101.7)_ = 19.23; *p* < 0.0001, main effect of drug: *F*_(1,38)_ = 24.21; *p* < 0.0001; genotype × drug: *F*_(1,38)_ = 1.11; *p* = 0.30; three-way RM ANOVA). ***E***, The number of spikes fired in gbz was also similar between genotypes (main effect of genotype: *F*_(1,19)_ = 0.01; *p* = 0.90, main effect of stim intensity: *F*_(1.57,29.83)_ = 6.96; *p* < 0.01, interaction: *F*_(8,152)_ = 0.69; *p* = 0.70; two-way RM ANOVA).

We hypothesized that inhibitory dysfunction would lead to larger postsynaptic potentials (PSPs) and increased firing rates in Het neurons. To the contrary, we found that PSPs in Het and WT neurons, evoked by a single stimulation of SC axons (Fig. 2*A*), had statistically equivalent peak amplitudes across a broad range of stimulus intensities (two-way RM ANOVA; stimulus intensity: *p* < 0.0001; genotype: *p* = 0.44; intensity × genotype interaction: *p* = 0.36; Fig. 2*B*). While the lack of difference between genotypes was contrary to our original hypothesis, it is possible that other changes within the circuit such as homeostatic plasticity could compensate for decrements in inhibition. Thus, we repeated this experiment comparing synaptic responses with inhibition blocked with gabazine (gbz; 10 μm) to measure isolated excitatory input. Our expectation was that if excitation was scaled downward in Het neurons, WT neurons would exhibit larger responses than Het when inhibition was blocked. Instead, bath application of gabazine increased the postsynaptic responses of both genotypes equivalently. PSPs amplitude (measured as area under the curve with spikes truncated because peak amplitude is confounded by spikes; Fig. 2*C*), firing probability (Fig. 2*D*), and number of spikes (Fig. 2*E*) increased in both Het and WT neurons, with no significant differences between genotypes. Thus, this experiment revealed that activation of the hippocampal microcircuit produces similar responses in CA1 pyramidal neurons despite *Scn1a* haploinsufficiency.

### Temporal integration of theta burst activity is unaltered in *Scn1a* haploinsufficient CA1

**Figure 3.**
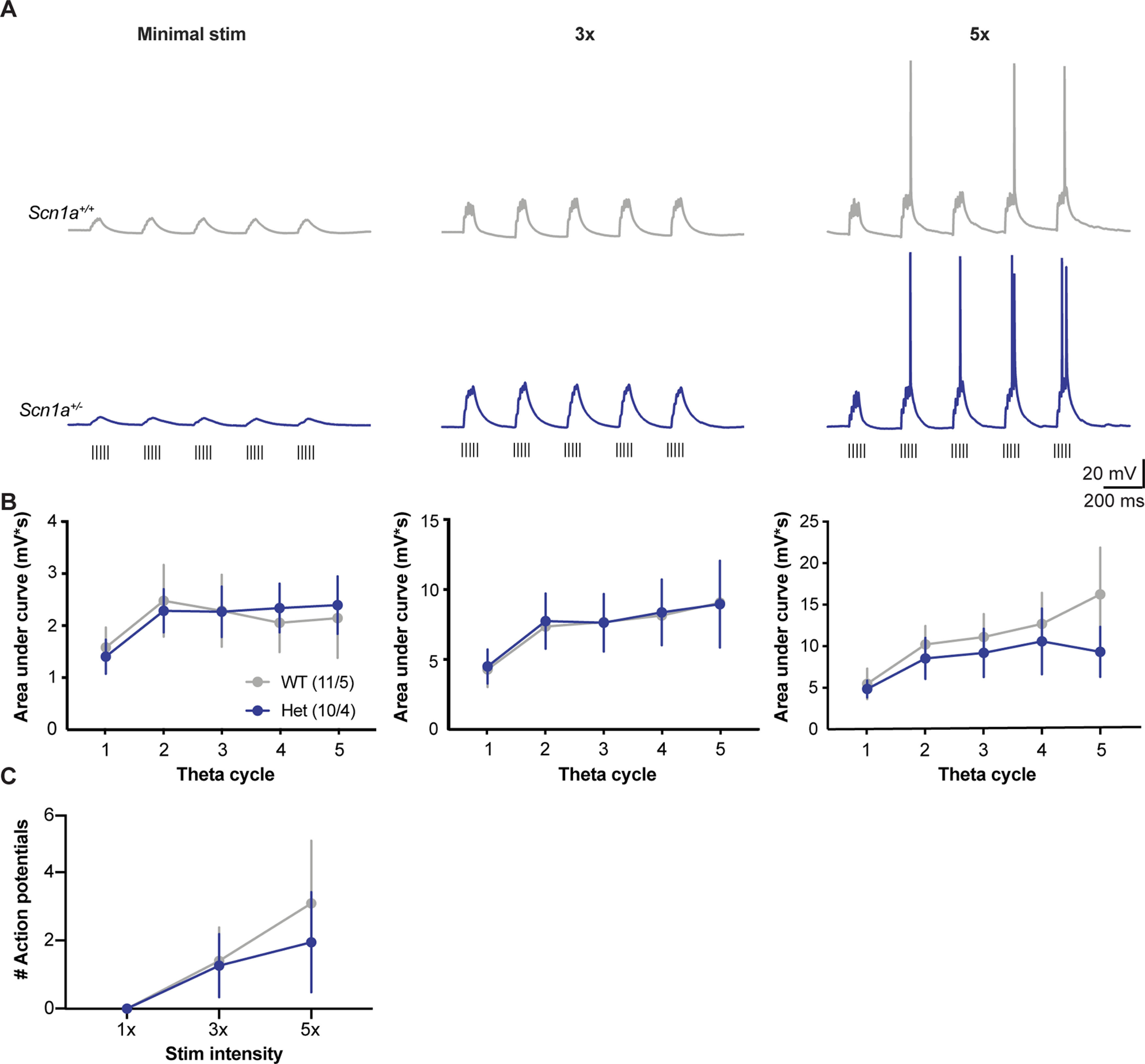
Normal temporal synaptic integration in *Scn1a*^+/−^ neurons. ***A***, Example PSPs recorded in CA1 pyramidal cells from *Scn1a^+/+^* (top, gray) and *Scn1a*^+/−^ (bottom, blue) mice in response to theta burst stimulation (hash marks below) of SC axons at 1×, 3×, and 5× the minimal stim intensity with no synaptic blockers. ***B***, No change in amplitude of PSPs, measured as area under the curve, between genotypes [main effect of genotype: *F*_(1,19)_ < 0.41; *p* > 0.53; *n* = (cells/mice)]. ***C***, No difference in number of action potentials fired in response to theta stimulation between genotypes (main effect of genotype: *F*_(1,19)_ = 0.06; *p* = 0.81, main effect of stim intensity: *F*_(1.04,19.82)_ = 4.40; *p* = 0.048, interaction: *F*_(2,38)_ = 0.19; *p* = 0.83; two-way RM ANOVA).

While decreased interneuron excitability did not alter the ability of inhibition to gate activation of CA1 pyramidal neurons in response to single SC stimulation, we hypothesized that physiological patterns of activity might cause a breakdown of inhibition and reveal underlying circuit hyperexcitability. We tested this hypothesis by holding neurons in current clamp and stimulating the SC tract with theta bursts (five bursts, five stimuli/burst at 100 Hz, 200-ms intraburst interval; Fig. 3*A*). Theta burst stimulation was repeated at 1×, 3×, and 5× the threshold for evoking reliable responses. The responses of Het neurons were statistically indistinguishable from those of WT neurons. PSP amplitudes for the temporally summed PSPs from each burst (measured as area under the curve with action potentials truncated) were unchanged between genotypes across theta cycles, at each stimulus intensity measured (main effect of genotype: *F*_(1,19)_ < 0.41; *p* > 0.53; *n* = 11 WT; 10 Het; Fig. 3*B*). Number of action potentials evoked was also unchanged between genotypes across theta cycles, at each stimulus intensity measured (Fig. 3*C*). This suggests that even when stimulated with repeated high frequency inputs designed to mimic ongoing physiological activity, GABAergic inhibition was fully functional at controlling CA1 pyramidal neuron activity despite known changes to interneuron physiology caused by *Scn1a* haploinsufficiency.

### Reduced feedforward inhibition onto Het CA1 pyramidal neurons

**Figure 4.**
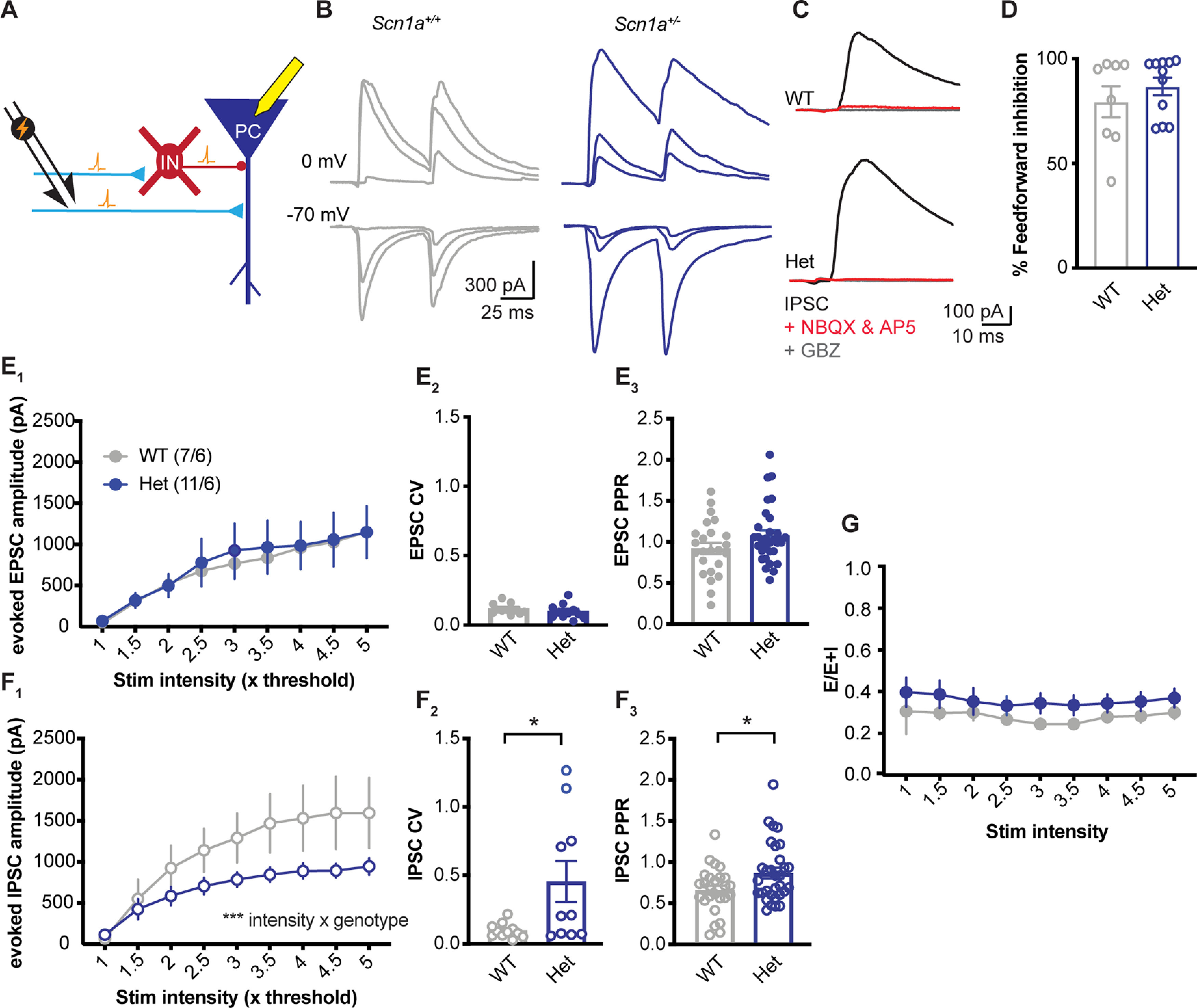
Reduced feedforward inhibitory input to *Scn1a*^+/−^ CA1 pyramidal neurons. ***A***, Experimental design. We performed whole-cell voltage-clamp recordings of CA1 pyramidal cells. SC axons were stimulated upstream from the CA1 pyramidal neuron, which directly release glutamate onto the CA1 pyramidal neuron to generate an EPSC, and onto inhibitory interneurons that then provide GABAergic IPSCs to the pyramidal cells. ***B***, Example traces of EPSCs (recorded at E_gaba_ = −70 mV) and IPSCs (recorded at E_glutamate_ = 0 mV) in WT (left, gray) and *Scn1a*^+/−^ (right, blue) neurons evoked at the minimal stimulation intensity required to evoke an EPSC (1×), 3×, and 5× the minimal intensity. ***C***, Example traces of IPSCs recorded in ACSF (black), in the presence of glutamate blockers (10 μm NBQX, 50 μm D-AP5, red) and in the presence of glutamate blockers + gabazine (10 μm, gray), demonstrating that the majority of the GABAergic input using this paradigm was feedforward inhibition. ***D***, 79.4% in WTs; 86.7% in *Scn1a*^+/−^; *p* = 0.38, *t* test. ***E_1_***, Peak amplitude of ESPCs by stimulation intensity is similar between genotypes [main effect of genotype: *F*_(1,16)_ = 0.03; *p* = 0.87, main effect of stimulation intensity: *F*_(1.158,18.53)_ = 17.45; *p* < 0.001, interaction: *F*_(8,128)_ = 0.13; *p* = 0.99; two-way RM ANOVA *n* = (cells/mice)]. ***E_2_***, The CV measured using 50 stimulations at 2.5× the minimal stim intensity (*p* = 0.33; *n* = 11 WT and *n* = 11 *Scn1a*^+/−^) and (***E_3_***) and PPR of EPSCs (*p* = 0.11; *n* = 24 WT and *n* = 31 *Scn1a^+/^*; *t* test) are not different between WT and *Scn1a*^+/−^ neurons. ***F_1_***, The IPSC growth curve is reduced in *Scn1a*^+/−^ neurons compared with WT (main effect of genotype: *F*_(1,16)_ = 3.03; *p* = 0.10, main effect of stimulation intensity: *F*_(1.158,18.53)_ = 39.11; *p* < 0.001, interaction: *F*_(8,128)_ = 4.10; *p* < 0.001). ***F_2_***, The CV of IPSCs is increased in *Scn1a*^+/−^ neurons (*p = 0.02; *n* = 11 WT and *n* = 11 *Scn1a*^+/−^), and (***F_3_***) PPR is increased (*p = 0.02; *n* = 26 WT and *n* = 31 *Scn1a*^+/−^). ***G***, Excitatory to inhibitory ratio, measured as the [area of the EPSC/(area of EPSC + area of IPSC)] was similar in WT and *Scn1a*^+/−^ neurons across stim intensities (main effect of genotype: *F*_(1,16)_ = 1.29; *p* = 0.22, stimulation intensity: *F*_(1.158,18.53)_ = 0.83; *p* = 0.44, interaction: *F*_(8,128)_ = 0.11; *p* = 0.99; two-way RM ANOVA).

Our synaptic integration experiments suggested that synaptic inhibition was fully intact, despite well documented changes in interneuron excitability in this model (Yu et al., 2006; Tai et al., 2014; Rubinstein et al., 2015; Favero et al., 2018) Thus, we next sought to determine whether changes to inhibitory synaptic physiology could be compensating for changes to excitability. We first measured feedforward synaptic inputs from CA3 to CA1 via stimulation of the SC axons. In these experiments the stimulating electrode was placed relatively distant from the neurons being recorded. In this configuration, GABAergic interneurons were activated by synaptic excitation rather than direct depolarization by the stimulating electrode (Fig. 4*A*), as evidenced by typical cessation of all synaptic responses after addition of glutamate receptor blockers to the ACSF (Fig. 4*C*,*D*). We voltage clamped CA1 pyramidal neurons, using a cesium-based internal solution, alternately at the reversal potential of glutamate (0 mV) and GABA (−70 mV) to isolate excitatory and inhibitory responses. After identifying a threshold stimulus intensity that consistently evoked a minimal response, growth functions were recorded by measuring responses while increasing stimulus current across a broad range of intensities.

For excitatory synaptic responses, statistical comparisons of the amplitude of evoked EPSCs and the shape of their growth functions revealed no statistical differences between WT and Het neurons (main effect of genotype: *F*_(1,16)_ = 0.03; *p* = 0.87, stimulation intensity: *F*_(1.158,18.53)_ = 17.45; *p* < 0.001, interaction: *F*_(8,128)_ = 0.13; *p* = 0.99; two-way RM ANOVA; Fig. 4*E*). These data show that excitatory synaptic input is normal at this synapse in Het mice with no signs of compensatory plasticity (e.g., homeostatic scaling). In contrast, comparison of evoked IPSC between genotypes revealed a significant change in growth function shape for inhibitory synaptic input as measured by the significant interaction of stimulus intensity and genotype (main effect of genotype: *F*_(1,16)_ = 3.03; *p* = 0.10, stimulation intensity: *F*_(1.158,18.53)_ = 39.11; *p* < 0.001, interaction: *F*_(8,128)_ = 4.10; *p* < 0.001; two-way RM ANOVA; Fig. 4*F*). This result is in line with previous reports of decreased excitability in specific subtypes of hippocampal GABAergic interneurons. It is noteworthy, however, that while the interaction of these two variables that make up the growth function is significant, there was not a significant difference in IPSC amplitude between genotypes either overall or at any individual stimulation intensity (ascertained by *post hoc* Sidak’s multiple comparisons tests: *t* < 1.696; *p* > 0.73). This indicates that despite interneuron intrinsic excitability changes, synaptic inhibition is largely intact in response to single activations of SC axons driving activity through the hippocampal circuit. This is supported by the ratio of excitation to inhibition [EPSC/(EPSC + IPSC)] as a function of stimulus intensity, which revealed no significant changes to this balance even as the circuit was driven by stronger stimuli (main effect of genotype: *F*_(1,16)_ = 1.29; *p* = 0.22, stimulation intensity: *F*_(1.158,18.53)_ = 0.83; *p* = 0.44, interaction: *F*_(8,128)_ = 0.11; *p* = 0.99; two-way RM ANOVA; Fig. 4*G*).

We further examined isolated excitatory and inhibitory synaptic inputs to understand whether the underlying physiology of these synapses was normal or altered after loss of *Scn1a*. Synaptic vesicle release at excitatory synapses, measured by paired-pulse ratio (PPR) with an interstimulus interval of 50 ms, was unchanged (*p* = 0.11; *t* test; Fig. 4*E2*). Similarly, the coefficient of variation (CV) of EPSC amplitude was relatively low and equivalent across genotypes (*p* = 0.33; *t* test; Fig. 4*E3*). These data further indicate that presynaptic physiology of excitatory synapses is grossly normal in Het neurons despite decrements in inhibition and epilepsy caused by *Scn1a* haploinsufficiency.

The CV of IPSC amplitude measured in Het neurons was significantly increased compared with WT (*p* = 0.02; *t* test; Fig. 4*F2*). Increased CV is often indicative of a decrease in the number of presynaptic neurons, synapses, or active zones. The PPR for feedforward IPSCs was also significantly increased in Het neurons (*p* = 0.02; *t* test; Fig. 4*F3*). It should be noted that PPR for IPSCs in this experiment is confounded by the short-term plasticity of excitatory SC synapses that drive action potential firing in the GABAergic interneurons which then in turn causes synaptic release and IPSCs. Thus, different numbers of interneurons are potentially recruited by the first and second stimulus. As such, this measure of PPR does not reflect an isolated measure of the release properties of inhibitory synapses; direct stimulation of interneurons is necessary to measure short-term facilitation/depression of isolated inhibitory synapses.

**Figure 5.**
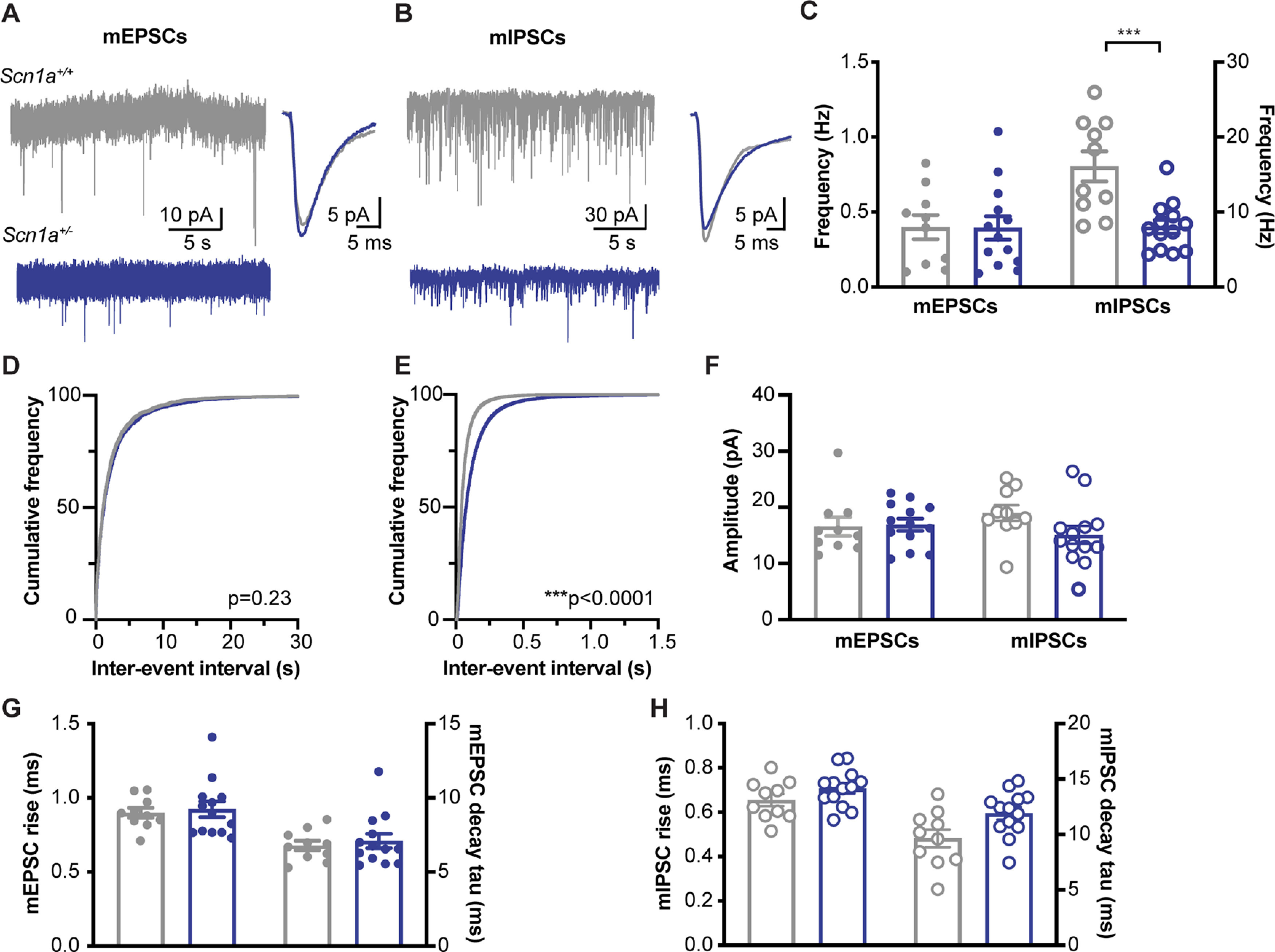
Reduced miniature IPSC frequency to *Scn1a*^+/−^ CA1 pyramidal neurons. ***A***, Example raw traces of mEPSCs, recorded in gabazine (10 μm) and 1 μm TTX (left) and averages (right). ***B***, Example mIPSCs recorded in glutamate blockers (10 μm NBQX, 50 μm D-AP5). ***C***, The frequency of mEPSCs in unchanged, but mIPSCs are reduced in *Scn1a*^+/−^ neurons (***p < 0.001; *t* test; *N* = 10 cells from 4 mice WT; *n* = 13 cells from 6 mice *Scn1a*^+/−^). ***D***, The lack of change in mEPSC frequency between WT and *Scn1a*^+/−^ neurons is demonstrated in the similar cumulative frequency of interevent intervals plot. ***E***, The cumulative frequency plot of mIPSCs is shifted to the right for *Scn1a*^+/−^ neurons (K-S test). The amplitude (***F***) and kinetics (***G***, ***H***) of mEPSCs and mIPSCs are similar between genotypes (*p* > 0.05; *t* test).

To gather more information about the specific properties of excitatory and inhibitory synaptic physiology in Het hippocampi, we isolated and recorded responses to spontaneous release of vesicles in CA1 pyramidal neurons (Fig. 5*A*). Miniature EPSCs (mEPSCs) were isolated by adding blockers of action potentials [tetrodotoxin (TTX); 1 μm] and synaptic inhibition (gbz; 10 μm) to the ACSF. Comparing Het with WT neurons, there were no differences in mEPSC frequency (Fig. 5*C*, mEPSC mean frequency: 0.40 ± 0.08 Hz WT; 0.39 ± 0.08 Het; *p* = 0.97; *t* test; Fig. 5*D*, cumulative frequency: *p* = 0.23, K-S test), amplitude (mean amplitude: 16.58 ± 1.66 pA WT; 16.90 ± 1.09 Het; *p* = 0.87; *t* test; Fig. 5*E*), rise time (*p* = 0.72; *t* test; Fig. 5*G*), or decay kinetics (*p* = 0.62; *t* test; Fig. 5*G*). As with eEPSCs above, these data indicate normal excitatory synaptic physiology and a lack of compensatory change at glutamatergic synapses.

Miniature IPSCs (mIPSCs) were isolated with TTX and glutamatergic blockers (NBQX: 10 μm, APV: 50 μm). mIPSC frequency was significantly reduced in Het neurons compared with WT (Fig. 5*C*, mIPSC mean frequency: 16.09 ± 2.00 WT; 8.04 ± 0.91 Het; *p* = 0.0007; *t* test; Fig. 5*E*, mIPSC cumulative frequency: *p* < 0.0001, K-S test). mIPSC amplitude (*p* = 0.09; Fig. 5*E*), rise time (*p* = 0.16; Fig. 5*H*), and decay kinetics (*p* = 0.09; *t* test; Fig. 5*H*) were unchanged between Het and WT neurons. Decreases to mIPSC frequency are often attributable to decreases in vesicle release probability or to the number of inhibitory synapses, which is supported by the increased IPSC CV and PPR reported above.

### Inhibition onto *Scn1a* Het CA1 pyramidal neurons is normal when interneurons are directly stimulated

**Figure 6.**
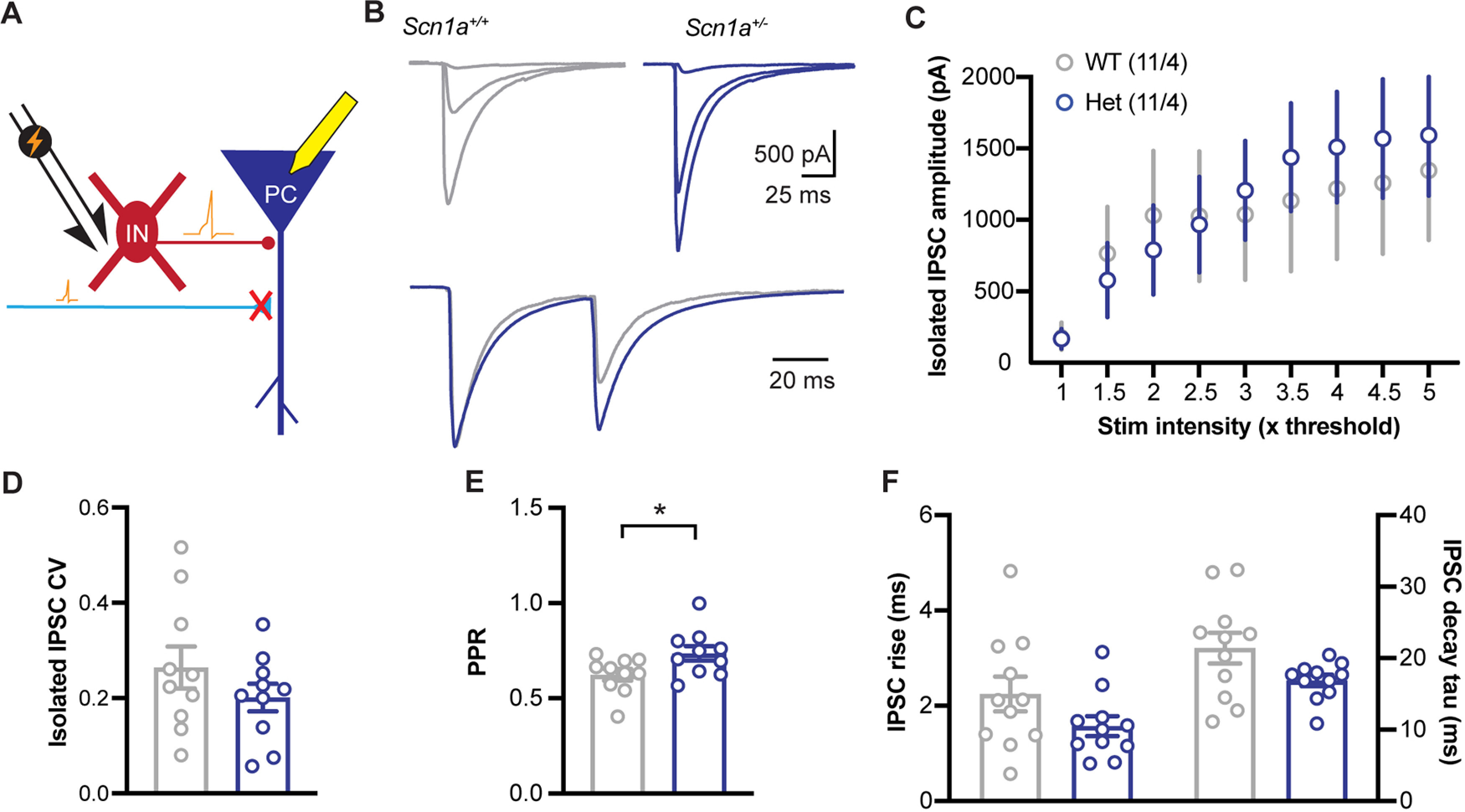
Direct activation of inhibitory interneurons reveals normal amplitude but more facilitating GABAergic responses in CA1 pyramidal neurons. ***A***, Experimental design. We stimulated near the recorded CA1 pyramidal cell (PC) in the presence of glutamatergic blockers (10 μm NBQX, 50 μm D-AP5) to directly activate local GABAergic interneurons (IN) and axons. ***B***, Example traces of IPSCs from WT (left, gray) and *Scn1a*^+/−^ (right, blue) neurons evoked at the minimal stimulation intensity required to evoke an EPSC (1×), 3×, and 5× the minimal intensity (top). Bottom, Overlaid paired-pulse stimulation (50-ms isi) traces recorded at 2.5× the minimal stim intensity, normalized to the peak of the first stimulation. ***C***, IPSC growth functions showed no deficits in *Scn1a*^+/−^ neurons (main effect of genotype: *F*_(1,20)_ = 0.03; *p* = 0.86, main effect of stim intensity: *F*_(1.63,32.60)_ = 15.09; *p* < 0.0001, interaction: *F*_(8,160)_ = 1.05; *p* = 0.40; two-way RM ANOVA). ***D***, No difference in CV of IPSCs measured using 50 stimulations at 2.5× the minimal stim intensity (*p* = 0.25, *t* test). ***E***, IPSC PPR is increased in *Scn1a*^+/−^ neurons (*p = 0.03; *t* test). ***F***, No difference in IPSC kinetics between genotypes (rise: *p* = 0.15; decay: *p* = 0.12; Mann–Whitney tests).

To examine more directly how inhibitory synaptic transmission is altered in Het neurons compared with WT, we performed voltage clamp experiments with the stimulus electrode positioned closer to the recording electrode to directly activate GABAergic interneurons and terminals rather than via excitatory synaptic stimulation of the interneurons. (Fig. 6*A*). IPSCs were then isolated with glutamate receptor blockers NBQX and APV in the ACSF. As above, IPSC growth functions were recorded by increasing stimulus strength in multiples of the threshold stimulus. Unlike in the indirect stimulation of inhibition experiments, IPSC growth functions showed no deficits in Het neurons (main effect of genotype: *F*_(1,20)_ = 0.03; *p* = 0.86, main effect of stim intensity: *F*_(1.63,32.60)_ = 15.09; *p* < 0.0001, interaction: *F*_(8,160)_ = 1.05; *p* = 0.40; two-way RM ANOVA; Fig. 6*B*,*C*). Also, unlike the direct stimulation experiments, there was no statistical change in IPSC amplitude CV between Het and WT neurons (*p* = 0.25; *t* test; Fig. 6*D*). IPSC PPR was increased in Het neurons compared with WT (*p* = 0.03; *t* test; Fig. 6*E*). No significant changes were revealed in IPSC kinetics (rise time: *p* = 0.15; decay tau: *p* = 0.12; Mann–Whitney tests; Fig. 6*F*). Overall, these data indicate that despite decreased excitability, *Scn1a* Het interneurons can be recruited by direct stimulation to activate across a broad range of stimulus intensities, resulting in normal levels of synaptic inhibition. Decreased synaptic inhibition growth functions in indirect stimulation experiments (Fig. 4) may thus be a result of decreased synaptic excitation to action potential input/output functions in interneurons. In Het neurons, inhibitory synapses do show an increase in PPR, suggesting a decrease in baseline probability of release that results in a decrease in short-term synaptic depression (which is not caused by changes in kinetics causing temporal summation).

### Inhibitory synaptic transmission is stable across frequencies in Het neurons

The data so far reveal that isolated inhibitory synaptic transmission was capable of controlling activity during theta stimulation of SC afferents. We hypothesized that circuit hyperexcitability could be the result of diminished intrinsic excitability in interneurons and inhibitory strength, which would be unmasked when interneurons were driven to fire at higher frequencies and/or for longer durations. To test this hypothesis, we measured isolated synaptic inhibition across theta bursts and during longer stimulus trains of varying frequency. In these experiments excitatory synaptic transmission was pharmacologically blocked with NBQX and APV and the stimulation electrode placed close enough to directly activate interneurons and GABAergic terminals synaptically linked to the CA1 pyramidal neuron from which we recorded, as in Figure 6*A*.

**Figure 7.**
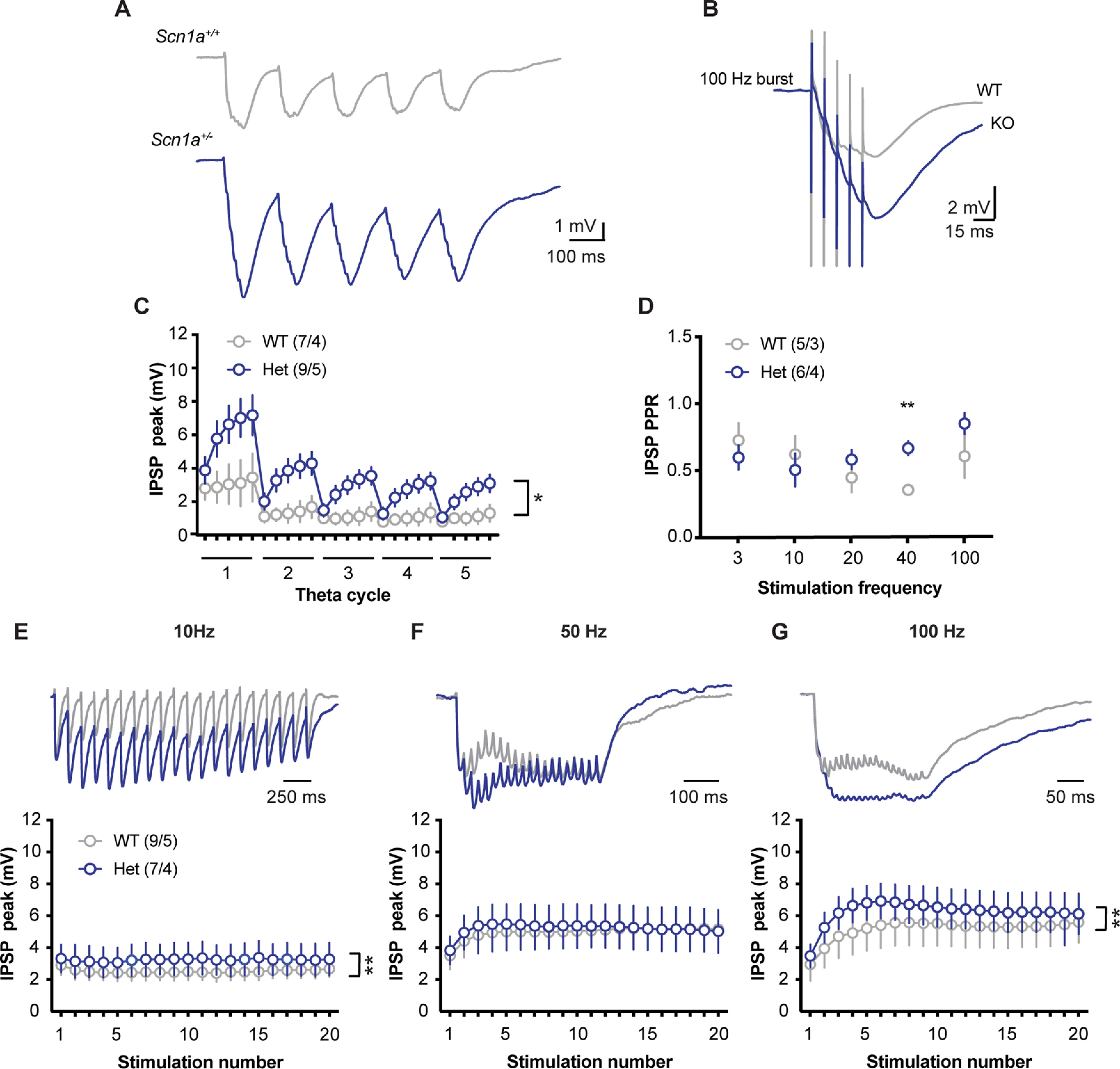
IPSPs are less depressing in *Scn1a*^+/−^ animals and provide increased inhibition at higher frequency stimulation. ***A***, Example traces of IPSPs in response to theta-burst stimulation from WT (top, gray) and Het (bottom, blue) neurons. ***B***, Overlaid traces of the first burst from the theta burst paradigm demonstrating that responses in WT neurons were much more depressing than those from *Scn1a*^+/−^ neurons. Stim intensity was normalized to generate the same amplitude (∼2 mV) for the first IPSP across cells. ***C***, IPSP maximum hyperpolarization for each stimulation in the theta burst paradigm in increased in *Scn1a*^+/−^ neurons [main effect of genotype: *F*_(1,14)_ = 6.00; *p = 0.03, main effect stim number: *F*_(1.60,22.38)_ = 18.51; *p* < 0.0001, interaction: *F*_(24,336)_ = 3.00; *p* < 0.0001; two-way RM ANOVA; *n* = (cells/mice)]. ***D***, PPR of IPSPs (amplitude of second IPSP/first IPSP) across a range of stim intensities showing that IPSPs are less depressing in *Scn1a*^+/−^ neurons at higher frequencies (main effect of genotype: *F*_(1,9)_ = 1.18; *p* = 0.31; main effect frequency: *F*_(1.69,15.17)_ = 2.03; *p* = 0.17, interaction: *F*_(4,36)_ = 2.29; *p* < 0.08; two-way RM ANOVA; **p < 0.01 Sidak’s multiple comparisons test). ***E***, IPSP peak hyperpolarization in response to 20 stimulations at 10 Hz (main effect of genotype: *F*_(1,280)_ = 8.86; **p = 0.003, stim number: *F*_(19,280)_ = 0.02; *p* > 0.99, interaction: *F*_(19,280)_ = 0.01; *p* > 0.99; two-way RM ANOVA), (***F***) 50 Hz (main effect of genotype: *F*_(1,280)_ = 0.41; *p* = 0.52, stim number: *F*_(19,280)_ = 0.18; *p* > 0.99, interaction: *F*_(19,280)_ = 0.02; *p* > 0.99), and (***G***) 100 Hz (main effect of genotype: *F*_(1,280)_ = 7.49; **p = 0.006, stim number: *F*_(19,280)_ = 0.58; *p* = 0.92, interaction: *F*_(19,280)_ = 0.03; *p* > 0.99).

Contrary to our hypothesis, hyperpolarization of the membrane was increased in response to theta burst stimulation of synaptic inhibition in Het neurons relative to WT controls (Fig. 7*A–C*). We adjusted the stimulation intensity to make the initial IPSP of the burst similar in amplitude (∼ 2 mV) across cells. Subsequent stimulations resulted in a significantly greater degree of hyperpolarization in Het neurons across the theta cycle (main effect of genotype: *F*_(1,14)_ = 6.00; *p = 0.03, main effect stim number: *F*_(1.60,22.38)_ = 18.51; *p* < 0.0001, interaction: *F*_(24,336)_ = 3.00; *p* < 0.0001; two-way RM ANOVA; Fig. 7*C*). This finding is consistent with the change to PPR illustrated in Figure 6*E* and is suggested by the decrease in mIPSC frequency in Figure 5*C*, which indicates that less short-term depression will occur during bursts of activity. The lack of change to IPSC rise and decay kinetics (Fig. 5*H*), as well as to input resistance and membrane time constant of CA1 pyramidal neurons (Table 1) across genotypes, in combination with these data suggest that increased temporal summation of IPSPs in Het neurons results from a change in presynaptic release, rather than postsynaptic receptor or membrane properties.

To further test the durability of inhibition in this system we extended our stimulus paradigm to trains of 20 stimuli, with stimulus frequencies of 10, 50, and 100 Hz (Fig. 7*E–G*). Synaptic inhibition was robust during these prolonged bursts in both WT and Het neurons. Het neurons exhibited small but statistically significant increases in peak hyperpolarization across the bursts at stimulus frequencies of 10 and 100, but not 50 Hz. These data were recorded in current clamp, and thus do not completely rule out the contribution of activation/deactivation of voltage-gated membrane channels. However, neurons of both genotypes were held at equivalent membrane potentials before stimulation and were recorded using the same internal and external solutions, and summed IPSPs are relatively small in magnitude. Taken together, these data indicate that synaptic inhibition onto CA1 pyramidal neurons is not compromised in Het neurons despite well-documented decrements in excitability in major subtypes of hippocampal GABAergic interneurons in *Scn1a* haploinsufficient mouse models.

## Discussion

The purpose of these experiments was to gain a deeper understanding of the link between *SCN1A* mutation, loss of function of the voltage-gated sodium channel Na_v_1.1, and the changes in neural activity and processing that lead to the seizures and cognitive deficits present in *SCN1A*-linked epilepsy disorders. We used a *Scn1a* Het mouse model of DS/epileptic encephalopathy and measured excitatory and inhibitory synaptic transmission and synaptic integration in CA1 hippocampal pyramidal neurons. While we originally hypothesized that hypoexcitability of hippocampal GABAergic interneurons, previously reported at this developmental stage in similar mouse models (Yu et al., 2006; Rubinstein et al., 2015; Almog et al., 2021), would cause network hyperactivity in response to physiologically patterned circuit input and/or compensatory decreases in excitatory synaptic signaling, our data revealed neither of these. Instead, our major findings consist of statistically significant, but relatively subtle, decreased feedforward inhibition, decreased probability of GABAergic synaptic release, and increased IPSP temporal summation in *Scn1a* Het neurons compared with WT, with a surprising lack of changes to excitatory synaptic physiology and temporal synaptic integration during theta burst activation.

Na_v_1.1 localizes to somata and dendrites throughout the brain (Westenbroek et al., 1989; Gong et al., 1999), but has been shown to cluster and control action potential generation primarily in GABAergic interneurons (Ogiwara et al., 2007). Gain of function mutations of *SCN1A* are associated with migraine (Dichgans et al., 2005), while loss of function mutations are associated with a broad range of epilepsy syndromes, ranging from the more mild GEFS+ to severe epileptic encephalopathies, including DS (Scheffer and Nabbout, 2019). *SCN1A* loss of function animal models, including *Drosophila*, zebrafish, mice, and rats, phenocopy many aspects of DS, like early life pharmacoresistant seizures, wide-ranging neurologic deficits, and high levels of early death (Schutte et al., 2016; Griffin et al., 2018). A common thread across models is the cell physiology phenotype of decreased action potential firing of forebrain GABAergic interneurons (Catterall, 2018). Information processing in forebrain microcircuits involves finely timed and balanced excitation and inhibition. Thus, we initially hypothesized that decreased inhibition would result in altered synaptic integration in *Scn1a* Het CA1 pyramidal neurons following activation of the hippocampal circuit with physiologically relevant patterned stimuli. However, our findings quite clearly show that both subthreshold and suprathreshold responses to theta burst protocols were normal in Het pyramidal neurons across a range of stimulus intensities.

While we did not observe the expected changes to synaptic integration of excitation and inhibition, our recordings did reveal changes in GABAergic inhibitory synaptic transmission in the Het hippocampus. We found a decrease in mIPSC frequency and increases in evoked IPSC PPR and CV when measuring feedforward inhibition, which could be caused by a decrease in the number of inhibitory synaptic inputs and/or a decrease in GABA release probability. While we cannot rule out a decrease in the number of inhibitory synapses, we found that ESPC amplitude and CV normalized when we directly stimulated GABAergic interneurons and terminals, while PPR remained elevated, indicating decreased probability of presynaptic vesicle release at interneuron-CA1 pyramidal neuron synapses. A functional outcome of the decrease in release probability is a reduction in short-term depression, with a net result of increased temporal summation of IPSPs across bursts or trains of synaptic activation. This provides a mechanism by which a reduction in GABA release probability could counteract or even overcome decrements in inhibitory neuron excitability, resulting in normalized synaptic integration during patterns of network activation. While it seems counterintuitive that a decrease in GABAergic release probability could be “compensatory,” i.e., protective or corrective in epilepsy, it has the functional effect of increasing the strength of inhibition during physiological patterns of activity. Indeed, GABA release probability is different at synapses onto pyramidal neurons in different brain regions and even across the dorsal-ventral (i.e., septo-temporal) axis of the hippocampus (Milior et al., 2016). Reduced release probability reduces synaptic depression, which can allow for more efficient inhibition during bursts of activity (González et al., 2014). Studies using other mouse models of epilepsy have reported similar reductions in GABA release probability resulting in reduced short-term depression in dentate gyrus granule cells in pilocarpine-induced temporal lobe epilepsy (Zhou et al., 2009; Faria and Prince, 2010), and in cortical pyramidal neurons in models of cortical dysplasia (Zhou et al., 2009; Faria and Prince, 2010) and posttraumatic epilepsy (Zhou et al., 2009; Faria and Prince, 2010). Notably, we cannot directly attribute the mechanism for the changes in synaptic release to *Scn1a* haploinsufficiency (i.e., to a decrease in Na_v_1.1 channels). Modulation of calcium channel expression has been reported as a mechanism for epilepsy-induced changes in GABA release (Faria et al., 2012). Thus, changes to GABAergic synaptic release may be a common compensatory mechanism induced in epilepsy or other disorders to balance excitation and inhibition.

Because our data showed that synaptic integration was unchanged in *Scn1a* Het CA1 pyramidal neurons despite interneuron hypoexcitability, we hypothesized that compensatory changes to excitatory synaptic transmission might be occurring in this model. Pyramidal neurons are well-known to have the capacity to change synaptic strength (Turrigiano, 2012) and intrinsic excitability (Remy et al., 2010) in response to long-term changes in activity. In mouse models of epilepsy, excitatory synaptic downscaling (H. Sun et al., 2013; Howard et al., 2014), upscaling (Avramescu and Timofeev, 2008; Houweling et al., 2005), and decreased intrinsic excitability (Howard et al., 2014) have been reported in CA1 pyramidal neurons. A recent study found increased synaptic strength of glutamatergic cortical inputs to dentate gyrus granule cells in the same *Scn1a* het mouse models used in our study (Mattis et al., 2022). Contrary to our hypothesis, our data did not reveal evidence of compensatory changes to synaptic or intrinsic excitability in *Scn1a* Het CA1 pyramidal cells. This may indicate that altered circuit activity does not reach thresholds for activation of these mechanisms, or that other factors may counterbalance or interfere with the expression of these changes.

While our theta burst synaptic integration experiments suggest that inhibition is functionally strong enough to balance excitation, this does not mean that interneurons in our study were without the well-documented excitability deficits. Our isolated recordings of feedforward IPSCs show clear deficits in response amplitude. When stimulating inhibition onto CA1 pyramidal neurons indirectly, via activation of excitatory SC axons onto interneurons which then release GABA onto the pyramidal neuron, feedforward inhibition was weaker in Het neurons compared with WT. Conversely, when inhibition was stimulated directly, by blocking excitatory transmission and placing the stimulus electrode where interneurons and GABAergic axons and terminals could be directly depolarized, inhibitory strength was equivalent between genotypes. A major difference between these experiments is that indirect stimulation requires synaptic integration, spike initiation at the axon initial segment, and axonal spike propagation by interneurons, each of which could be susceptible to decreased fidelity because of decreased sodium channel activity in Het interneurons. While synaptic transmission and axonal action potential propagation fidelity have not yet been measured directly in *Scn1a* Het models, several reports describe a depolarization of spike threshold in various populations of Het interneurons in *ex vivo* slice preparations (Tai et al., 2014; Rubinstein et al., 2015; Favero et al., 2018; Goff and Goldberg, 2019), but not in dissociated neurons presumed to be interneurons (Yu et al., 2006). Direct extracellular electrical stimulation of interneurons and GABAergic terminals eliminates synaptic integration and may be strongly suprathreshold, thus masking changes in spike initiation and/or propagation.

Our documentation of synaptic physiology in pyramidal neurons adds further complexity to an already complicated hypothesis of the causal role of inhibitory dysfunction in DS. Interestingly, the intrinsic hypoexcitability of parvalbumin-expressing and somatostatin-expressing interneurons originally discovered in Na_v_1.1 haploinsufficient mice at age P21 (Tai et al., 2014) normalizes in parvalbumin-positive and hippocampal stratum oriens (largely somatostatin-expressing) interneurons by P35–P56 (Favero et al., 2018; Almog et al., 2021). While mortality is at its peak in the earlier period correlating with greatest interneuron dysfunction, seizures and other deficits persist even in Het mice that survive long into adulthood (Yu et al., 2006; Han et al., 2012; Kalume et al., 2013). Our experiments bridged the age range with the most severe mortality (ranging from P21 to P38). Thus, we sampled from both mice that were likely to die of their epilepsy and those that would have survived long-term. That our data show that inhibitory synaptic transmission remained effective even at relatively high frequencies across extended trains in Het neurons suggests that inhibitory control of neural signaling is intact during most ongoing activity, even when interneurons are hypoexcitable and in mice with the most severe disease. This supports previous work in which *in vivo* recordings of both cortical network activity and identified interneurons were normal in anesthetized and awake Het mice during non-seizure (i.e., interictal) periods in both the severe disease state when interneurons are hypoexcitable (De Stasi et al., 2016; Tran et al., 2020) and at older ages when mortality is low and interneuron excitability has normalized (De Stasi et al., 2016; Tran et al., 2020).

Our findings leave open the questions of where and how the restraints on activity and processing of information by neural circuits break down and result in the seizures and cognitive deficits present in DS. While inhibitory interneurons exhibit clear hypoexcitability, we found no disruption of inhibition or synaptic integration during “physiological” theta bursts or even extended bursts of stimulation. This shows us that the link between mutation, cellular physiology, and circuit activity is not necessarily direct or intuitive. It may be that our hypotheses and expectations of results are simply incorrect, that our experimental parameters do not cover broad enough ranges, or that our methodologies are not subtle enough to detect nuanced changes. What is clear is that understanding the cell and circuit mechanisms of complex neurologic disorders such as DS requires a holistic understanding of direct and indirect changes to the intrinsic and synaptic physiology of many different neuron subtypes and their interactions.

## Synthesis

Reviewing Editor: Viji Santhakumar, University of California Riverside Decisions are customarily a result of the Reviewing Editor and the peer reviewers coming together and discussing their recommendations until a consensus is reached. When revisions are invited, a fact-based synthesis statement explaining their decision and outlining what is needed to prepare a revision will be listed below. The following reviewer(s) agreed to reveal their identity: Allyson Alexander, John Gray.

The manuscript presents a carefully examination of an intrinsic and synaptic electrophysiological characteristics in CA1 pyramidal cells in mice with Scn1a haploinsufficiency, an established mouse model of Dravet syndrome (DS). Based on prior studies, the authors proposed that decreased intrinsic excitability of GABAergic interneurons in Scn1a haploinsufficiency, reported previously, would alter synaptic integration in the hippocampus. Surprisingly, they show that synaptic integration and E/I balance are remarkably unaltered suggesting possible compensatory mechanisms. The reduction in mIPSC frequency is consistent with prior studies. Interestingly, they found evidence for decrease release probability at inhibitory synapses onto CA1 pyramidal cells that paradoxically could normalize inhibition during bursts or trains of activity by allowing enhanced synaptic facilitation. The reviewers agree that the paper is interesting, timely, and carefully performed. A few revisions and clarifications would strengthen this study.

1. Some of the results here are inconsistent with a number of other studies that have established that SCN1A mutation results in deficits in GABAergic inhibition and decrease in synaptic inhibition. This warrants more extensive discussion drawing contrasts with existing literature and would help in integrating different findings in seemingly similar models. While not necessarily inclusive, some examples of potential aspects of discussion are included below

a. Given the evidence for topological differences across the septo-temporal axis of the hippocampus, is there potential contribution of the region (Dong et al., 2009; Fanselow and Dong 2010; Malik et al. 2016), “middle of the dorsal-ventral axis of the hippocampus”, examined. There is also data that suggests that GABA release may be higher in slices from VH rather than DH and while VH glutamate release probability at Schaffer collateral synapses is remarkably higher in VH compared to DH (Milior et al., 2016). Could recording in the intermediate hippocampus region explain the difference observed here in this paper compared to previously published data showing deficits in GABAergic inhibition and enhanced excitability in this DS model?

b. Could differences in background strain explain the variability between studies? Mistry et al. found a larger reduction in sodium current in dissociated hippocampal inter neurons in the Scn1a+/− mice bred to C57BL/6J (F1.Scn1a+/−) compared to Scn1a+/− mice on strain 129S6/SvEvTac (129.Scn1a+/−) C57BL/6J. Miller et al., 2013 also show that background strain could be responsible for phenotype variability. Could the genetic background of this particular mouse model of DS explain the differences observed in the electrophysiological data presented here? Please provide some detail or discuss difference between mice used in these particular experiments compared to Mistry et al. and Miller et al.

2. The title needs to be rephrased to better reflect the conclusions.

3. In Figure 4F illustrates a significant effect of intensity and interaction with genotype but not an effect of genotype. Was the pairwise comparison at 3X and above significant?

4. Authors show that CV of IPSC amplitude and IPSC PPR measured in Het neurons was significantly increased compared with WT while mIPSC frequency is decreased in Het neurons (Fig. 4F2 and F5C). As authors suggests, this indicates that there is a decrease release probability and/or the number of presynaptic neurons, synapses, or active zones. Immunohistochemistry staining for the vesicular GABA transporter (VGAT) in hippocampal slices of both Nav1.1 (heterozygous) mutant and WT might provide further support for a specific reduction in release probability rather than a decrease in number of inhibitory interneuron or inhibitory synapses. Minimally, the authors should discuss the possibility that there may also be concurrent reduction in inhibitory synapses associate with changes in release probability.

5. The evidence for reduced release probability at inhibitory synapses is not highlighted in the abstract and should be included and discussed.

6. Additional minor edits

a. Typo in final sentence of significance statement: “found that changes GABA release probability”

b. In the introduction authors state “Despite clear evidence of severe epilepsy, broad neurological dysfunction, and early death in this mouse model..”. Please cite the papers that used this particular mouse line to show severe epilepsy and neurological dysfunction.

c. Figure 2 sample traces for both ACSF and with Gabazine suggests that there is decreased firing probability in Het mice compared to WT controls. Perhaps better sample traces that corresponds to averages should be provided.

d. Please check for inclusion of sample sizes which is missing for data related to Fig .3

e. Figure 3B - in the left two panels could be presented at a different scale to reduce overlap and visualize the groups.
